# Genome sequence of *Fusarium oxysporum* strain ByF01, the causal agent of root rot of *Knoxia roxburghii* in China

**DOI:** 10.1186/s12863-024-01242-w

**Published:** 2024-06-14

**Authors:** Chunju Liu, Zhaohui Guo, Lei Zhang, Jiahong Dong, Xiahong He, Heng Li, Bin Qiu

**Affiliations:** 1 Institute of Medicinal Plant Cultivation, School of Chinese Materia Medica, Academy of Southern Medicine, Yunnan University of Chinese Medicine, Kunming, 650500 China; 2 College of Plant Protection, Yunnan Agricultural University, Kunming, 650201 China; 3 Southwest Forestry University, Kunming, 650244 China; 4 R&D Center of Yunnan Yuntianhua Co., Ltd, Kunming, 650228 China

**Keywords:** Complete genome, *Fusarium oxysporum*, Root rot, *Knoxia roxburghii*

## Abstract

**Objectives:**

*Knoxia roxburghii* is a member of the madder (Rubiaceae) family. This plant is cultivated in different areas of China and recognized for its medicinal properties, which leads to its use in traditional Chinese medicine. The incidence of root rot was 10–15%. In June 2023, the causal agent of root rot on *K. roxburghii* was identified as *Fusarium oxysporum.* To the best of our knowledge, this is the first report of the complete genome of *F. oxysporum* strain ByF01 that is the causal agent of root rot of *K. roxburghii* in China. The results will provide effective resources for pathogenesis on *K. roxburghii* and the prevention and control of root rot on this host in the future.

**Data description:**

To understand the molecular mechanisms used by *F. oxysporum* to cause root rot on *K. roxburghii*, strain ByF01 was isolated from diseased roots and identified by morphological and molecular methods. The complete genome of strain ByF01 was then sequenced using a combination of the PacBio Sequel IIe and Illumina sequencing platforms. We obtained 54,431,725 bp of nucleotides, 47.46% GC content, and 16,705 coding sequences.

## Objective

*Knoxia roxburghii* (syn. *K. valerianoides*), known locally as ‘Zi Daji,’ is a perennial herb that is a member of the madder (Rubiaceae) family. It is cultivated in different areas of China and recognized for its medicinal properties in traditional Chinese medicine (TCM) [1]. However, root rot is a common disease that affects the cultivation of various root and rhizome medicinal plants, and it has been reported on numerous occasions [2, 3]. The incidence of disease was 10–15% in some *K. roxburghii* plantations. In June 2023, the pathogen of *K. roxburghii* root rot was identified as *Fusarium oxysporum* based on morphological and molecular methods [4].

*F. oxysporum* is one of the 10 most important economic fungal pathogens, with a high degree of host specificity. According to its specificity for host plant infection, *F.oxysporum* is divided into different specialized types [5]. *F. oxysporum* causes significant economic losses for disease in many medicinal and crop plants [6, 7], including *Adenophora capillaris*, *Polygonatum odoratum*, and *Pulsatilla koreana* among others [8–10]. Although the root rot of *K. roxburghii* is becoming increasingly serious, few studies have reported its prevalence. In particular, the genetic information of this fungus still remains unknown. In our study, the complete genome of strain ByF01 of *F. oxysporum* on *K. roxburghii* from China was sequenced using a combination of the PacBio Sequel IIe (Pacific Biosciences, Menlo Park, CA, USA) and Illumina (San Diego, CA, USA) sequencing platforms.

## Data description

Strain ByF01 was isolated from diseased *K. roxburghii* roots collected from Xiangyun County, Dali Bai Autonomous prefecture of Yunnan Province, China (25°25′N, 100°40′E), in 2021. This strain was identified as *F. oxysporum* based on morphological characteristics, PCR amplification [11], a phylogenetic analysis based on the nucleotide sequences of *cmdA*, *rpb2*, *tef1*, and *tub2*, and a pathogenicity test with 1-year-old healthy seedlings of *K. roxburghii*, which fulfilled Koch′s postulates [4]. Strain ByF01 was deposited in the Institute of Medicinal Plant Cultivation, Academy of Southern Medicine, Yunnan University of Chinese Medicine, Yunnan, China.

ByF01 was cultivated in potato dextrose broth (PDB) liquid medium at 28℃ and 150 rpm for 3 days. Genomic DNA was extracted using a fungal DNA extraction kit (magnetic beads) (Shanghai Majorbio Bio-pharm Technology Co., Ltd., Shanghai, China). The quality and concentration of DNA were determined by 1.0% agarose gel electrophoresis and a NanoDrop^®^ ND-2000 spectrophotometer (Thermo Fisher Scientific, Inc., Waltham, MA, USA). The complete genome of strain ByF01 was sequenced by Shanghai Majorbio Bio-pharm Technology Co., Ltd. using a combination of PacBio Sequel IIe and Illumina sequencing platforms. All of the analyses were performed using the free online platform of Majorbio Cloud Platform (https://cloud.majorbio.com). After sequencing, 110,926 raw reads and 2,400,749,764 raw bases were obtained, with a mean length of 21642.80 bp (Table 1, Data set 1 [18]), . The PacBio reads and Illumina reads were assembled using Hifiasm v. 0.19.5. The quality of genome assembly was assessed using the Core Eukaryotic Genes Mapping Approach (CEGMA) v. 2.5 and Benchmarking Universal Single-Copy Orthologs (BUSCO) v. 5.4.5 [12, 13]. The genomic coding genes and genomic repetitive sequences were predicted using Maker2 v. 2.32 and Repeat Masker v. 4.1.4, respectively [14, 15]. Barrnap v. 0.9 and tRNA-scan-SE v. 2.0.12 software were used to predict the rRNA and tRNA contained in the genome, respectively. BLAST + v. 2.3.0 was used to compare the predicted coding gene sequence within the NCBI database to obtain the corresponding functional annotation information. Clusters of Orthologous groups of proteins (COG), Gene Ontology (GO), Kyoto Encyclopedia of Genes and Genomes (KEGG), and Carbohydrate Active EnZymes (CAZy) were used for the functional protein analysis [14].

The genomic characteristics are shown in Table 1, Data file 1, Data set 1 [16, 18]. We obtained 54,431,725 bp of nucleotides and 47.46% GC content. A total of 16,705 coding sequences were identified, and the Repeat content was 0.11. There were 27, 4,602 and 6,109 scaffolds, KEGG, and COG, respectively. There were 330 transfer RNAs (tRNA) and 315 ribosomal RNAs (rRNA) among the non-coding RNAs.

**Table 1:** Overview of data files/data sets

Label	Name of data file/data set	File types (file extension)	Data repository and identifier (DOI or accession number)
Data file 1	Genome summary of *F. oxysporum* strain ByF01	Spreadsheet (.xls)	Figshare, 10.6084/m9.figshare.25609713 [16]
Data file 2	The functional prediction of *F. oxysporum* strain ByF01	Word(.doc)	Figshare, 10.6084/m9.figshare.25609713 [16]
Data file 3	The annotations in gff format of ByF01	(.gff)	Harvard Dataverse, 10.7910/DVN/OED35D [17]
Data set 1	Assembled genome	fastq files (.fastq)	NCBI Sequence Read Archive https://identifiers.org/ncbi/insdc.gca:GCA_034400335.1 [18]
Data set 2	Genome assembly of *F. oxysporum*	fasta file (.fna)	NCBI GenBank https://identifiers.org/ncbi/insdc.sra:SRP465557 [19]
Data set 3	Complete genome sequence	fasta file (.fasta)	NCBI Nucleotide https://www.ncbi.nlm.nih.gov/nuccore/JAXIVL000000000.1/ [20]

The results of the evaluation of assembled genome quality showed that the CEGMA and BUSCO analyses were 96.37 and 98.5% complete, respectively. In addition, an analysis of the database of fungal virulence factors (DFVF) (http://sysbio.unl.edu/DFVF/index.php10.1093/database/bas032) showed that ByF01 contained 1,606 pathogenic factors and 411 antibiotic resistance genes.

The results of functional annotation are shown in Table 1, Data file 2 [16]. A total of 6,109 proteins were successfully annotated in the COG database and divided into 25 categories [21] in which 1,042 proteins were predicted to function in carbohydrate transport and metabolism (G), and 867 proteins were found to be predicted for general function (R). A volume of 63.84% genes were successfully annotated into the GO database [22] and divided into three main functional categories, including biological process, cellular component, and molecular function. However, approximately half of the total genes were involved in molecular function. Most of the genes in molecular function were primarily annotated in catalytic activity, binding, transporter activity, and transcription regulatory activity. The KEGG analysis showed that the successfully annotated 7,745 genes that encoded proteins could be divided into six major functional categories and 47 secondary categories [23]. Metabolism (3,557) was the most abundant group, followed by human diseases (1,625), organismal systems (789), genetic information processing (771), cellular processes (634) and environmental information processing (369). The results of the CAZy comparison showed that the carbohydrate active enzyme gene annotation was divided into six types [24], including glycoside hydrolases (42.3%), carbohydrate esterases (20.27%), auxiliary activities (20.54%), glycosyl transferases (12.43%), polysaccharide lyases (3.38%), and carbohydrate-binding modules (1.08%) .

In this study, we obtained the draft genome assembly of *F. oxysporum* strain ByF01 and performed a bioinformatics analysis. To the best of our knowledge, this is the first report of the genome sequence of *F. oxysporum* strain ByF01 that causes root rot of *K. roxburghii* in China and throughout the world. The genomic data will provide effective resources to better understand the pathogenesis of *K. roxburghii* and prevent and control root rot on this TCM in the future.

## Limitations

There are many kinds of *F.oxysporum* in different hosts have been sequenced. So far, tomato *F.oxysporum* (*Fol*4287) is relatively complete. Although many contigs have been obtained this time, the genome size is smaller than that of *Fol*4287 (61.39 Mb). The reason for these variations is still unclear.

## Data Availability

The data described in this Data note can be freely and openly accessed on NCBI accession number GCA_034400335.1, SRA under SRA accession ID SRP465557, NCBI GenBank under accession number JAXIVL010000000, figshare (10.6084/m9.figshare.25609713), and Harvard Dataverse (10.7910/DVN/OED35D). Please see Table 1 for details and links to the data.

## Abbreviations

BUSCO: Benchmarking Universal Single-Copy Orthologs
CAZy: Carbohydrate Active EnZymes
CEGMA: Core Eukaryotic Genes Mapping Approach
COG: Clusters of Orthologous groups of proteins
GO: Gene Ontology
KEGG: Kyoto Encyclopedia of Genes and Genomes
NCBI: National Center for Biotechnology Information
